# Minimum follow-up time required for the estimation of statistical cure of cancer patients: verification using data from 42 cancer sites in the SEER database

**DOI:** 10.1186/1471-2407-5-48

**Published:** 2005-05-17

**Authors:** Patricia Tai, Edward Yu, Gábor Cserni, Georges Vlastos, Melanie Royce, Ian Kunkler, Vincent Vinh-Hung

**Affiliations:** 1 University of Saskatchewan, Faculty of Medicine, Saskatoon; Department of Radiation Oncology, Regina, Canada; 2 Radiation Oncology Program, London Regional Cancer Centre, University of Western Ontario, London, Ontario, Canada; 3 Bács-Kiskun County Teaching Hospital, Surgical Pathology, Kecskemét, Hungary; 4 Geneva University Hospitals, Department of Gynecology and Obstetrics, Senology and gynecologic oncology unit, Geneva, Switzerland; 5 University of New Mexico, Cancer Research and Treatment Center, Albuquerque, NM, USA; 6 Department of Clinical Oncology, Western General Hospital, Edinburgh, Scotland, UK; 7 AZ-VUB, Oncologisch Centrum, Jette, Belgium

## Abstract

**Background:**

The present commonly used five-year survival rates are not adequate to represent the statistical cure. In the present study, we established the minimum number of years required for follow-up to estimate statistical cure rate, by using a lognormal distribution of the survival time of those who died of their cancer. We introduced the term, threshold year, the follow-up time for patients dying from the specific cancer covers most of the survival data, leaving less than 2.25% uncovered. This is close enough to cure from that specific cancer.

**Methods:**

Data from the Surveillance, Epidemiology and End Results (SEER) database were tested if the survival times of cancer patients who died of their disease followed the lognormal distribution using a minimum chi-square method. Patients diagnosed from 1973–1992 in the registries of Connecticut and Detroit were chosen so that a maximum of 27 years was allowed for follow-up to 1999. A total of 49 specific organ sites were tested. The parameters of those lognormal distributions were found for each cancer site. The cancer-specific survival rates at the threshold years were compared with the longest available Kaplan-Meier survival estimates.

**Results:**

The characteristics of the cancer-specific survival times of cancer patients who died of their disease from 42 cancer sites out of 49 sites were verified to follow different lognormal distributions. The threshold years validated for statistical cure varied for different cancer sites, from 2.6 years for pancreas cancer to 25.2 years for cancer of salivary gland. At the threshold year, the statistical cure rates estimated for 40 cancer sites were found to match the actuarial long-term survival rates estimated by the Kaplan-Meier method within six percentage points. For two cancer sites: breast and thyroid, the threshold years were so long that the cancer-specific survival rates could yet not be obtained because the SEER data do not provide sufficiently long follow-up.

**Conclusion:**

The present study suggests a certain threshold year is required to wait before the statistical cure rate can be estimated for each cancer site. For some cancers, such as breast and thyroid, the 5- or 10-year survival rates inadequately reflect statistical cure rates, and highlight the need for long-term follow-up of these patients.

## Background

The normal distribution is often used to describe the random variation of data in many scientific disciplines. However some distributions are skewed with low mean values and large variance. The distributions may be exclusively positive, such as the duration of survival of cancer patients with chronic leukemias [1], the incubation time of infectious diseases [2], and the abundance of biological species [3], etc. These skewed distributions often fit the lognormal distribution [4-7]. A lognormal distribution is one with random variables whose logarithms follow a normal distribution. The lognormal survival time of cancer patients, who died of their disease, has been tested and applied for various anatomic sites [8-21].

The term survival rate is used commonly, yet it is inaccurate. Five- and ten-year survival rates are commonly used in the literature. Kaplan-Meier or life-table (actuarial) methods estimate the proportion (or fraction) of survivors. In this study, the term "survival rate" means "survival fraction" expressed in percentage, and the term "cure rate" means "cure fraction" expressed in percentage, to be proper.

It is difficult to know the statistical cure rate, which is an estimation based on statistical models [11,15], especially for slowly proliferating tumors. Because of this limitation, oncologists usually discuss survival in terms of 5-year or 10-year survival rates. In certain rapidly growing tumors, the cancer-specific survival rate [22] reaches a plateau within 5–10 years and approaches the statistical cure rate, which is the survival rate observed when no more risk of death from the disease. In the Kaplan-Meier method [23], a person with residual cancer but who died of reasons other than the specific cancer is censored for cancer-specific survival. In the present study we applied the analysis on the cancer-specific survival rates. The statistical cure was reached at the plateau of the Kaplan-Meier plot of the cancer-specific survival rate. For some fast proliferating cancers, such as pancreatic and stomach cancers, the plateau appears within 10 years. However for some slow proliferating cancers, such as thyroid and early breast cancers, the plateau does not appear even after decades. Hence the present commonly used five-year survival rates are not adequate to represent the statistical cure rate.

In the present study, we established the minimum number of years required for follow-up in order to estimate the statistical cure rate, by using a lognormal distribution of the survival time of those who died of their cancer by applying the result derived by Limpert *et al*.[24] This minimum number of years required for follow-up was defined as the threshold year by μ* × (σ*)^2 ^where μ* is the median and σ* is the multiplicative standard deviation of a lognormal distribution (see Additional file 1 : Appendix). The follow-up time for patients dying from the specific cancer covers most of the survival data, leaving less than 2.25% uncovered. This is close enough to cure from that specific cancer.

## Methods

### Data sources

We analysed the 1973–1999 Database of the Surveillance, Epidemiology, and End Results (SEER) Program [25] of the United States National Cancer Institute. Data from registries of Connecticut and Detroit were used in this study and results were compared with the SEER-9 registries. The cancer sites were chosen according to the SEER codes. Primary site, vital status, cause of death, and survival time information were used. Those patients with unknown or zero survival time and unknown cause of death were excluded.

To test for lognormality of survival time of cancer patients who died of cancer-specific disease, patients diagnosed from 1973–1992 in the registries of Connecticut and Detroit were chosen so that a maximum of 27 years was allowed for follow-up to 1999. A total of 49 specific cancer sites were tested. For prostate, salivary gland, breast, and thyroid cancer, patients diagnosed from 1973–1977 were chosen to allow a long enough time for follow-up to 1999. For cancer sites with high frequencies of older patients, such as lung and bronchus, colon, prostate, and breast, younger patient ages less than 60 were chosen, because elderly patients are more likely to die of intercurrent diseases and so affect the distribution of cancer-specific deaths versus other deaths.

As an illustration for special cohorts of interest, breast cancer was further analyzed based on accepted prognostic factors such as stage, histologic type and tumor grade, to show that the threshold years were different for different cohorts of interest.

### Statistical analysis

The survival times of the cancer patients who died of their disease were tested for goodness of fit for lognormality using a minimum chi-square method. The class intervals were in the powers of 2 in months of the survival time, such as 0–2, >2–4, >4–8, >8–16, and so on. The values of M, mean of the log (survival time), and S, standard deviation of the log (survival time), were varied in the tests so that a minimum chi-square value was obtained. The null hypothesis states that there is no difference between the observed data distribution and the lognormal distribution. It is rejected if P < 0.05. The values of M and S were obtained when the chi-square value reached a minimum.

**Figure 1:**
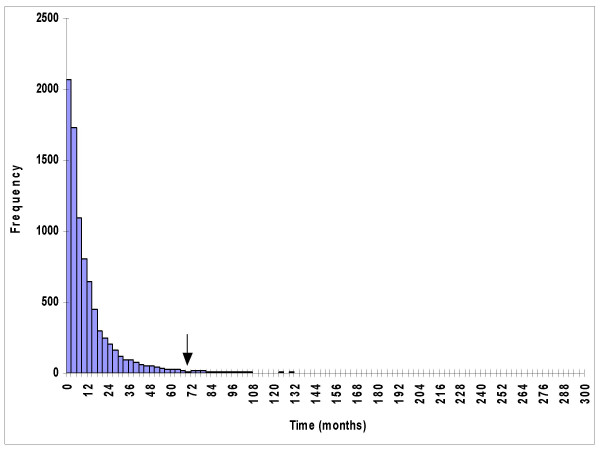
The survival time for cancer patients who died of their disease. The single arrow shows the location of the threshold year (τ). This diagram uses stomach cancer data as an example.

**Figure 2:**
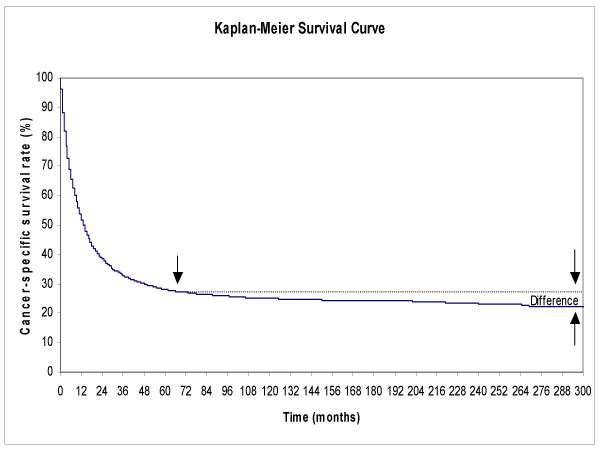
Cancer-specific survival rate of cancer patients who died of their disease calculated by Kaplan-Meier method. Note the small difference between the cancer-specific survival rate at τ year as compared to the long-term survival rate. The single arrow shows the location of the threshold year (τ). The pair of opposing arrows at the long-term follow-up shows that the difference between the estimated and actual cancer-specific rate is small, less than six percentage points. The survival time for cancer patients who died of their disease for stomach cancer is shown as an example.

Let τ be the threshold year at which statistical cure rate can be estimated, then the cancer-specific survival rate can be obtained by Kaplan-Meier method with follow-up time equal to τ. This cancer-specific survival rate at time τ was an estimation of the statistical cure rate of the disease. It was compared with the long-term cancer-specific rate calculated using Kaplan-Meier method with the actual long-term data available up to 1999 (Figures 1 and 2).

## Results

### Lognormality

The present study verified that, for 42 specific organ sites out of 49 cancer sites in the SEER database, the survival time of cancer patients who died of their disease followed different lognormal distributions approximately. For the cancer sites with cancer-specific survival time following lognormal distribution, the number of patients and values of S, multiplicative standard deviation, M, median and P at minimum chi-square are listed in Table 1. All the P-values in Table 1 are above 0.10.

The seven specific organ sites failed in the test for lognormality were: lip, oropharynx, rectosigmoid junction, rectum, testis, urinary bladder, and kidney & renal pelvis.

### Estimation of statistical cure rate

The threshold years validated for statistical cure were found to range from 2.6 years for pancreatic cancer to 25.2 years for cancer of salivary gland. For these 40 cancer sites with survival time followed a lognormal distribution, their cancer-specific survival rates were obtained by Kaplan-Meier method at the threshold year, and they were compared with their corresponding long-term survival rates with follow-up to 1999. Out of the 42 cancer sites with survival time followed a lognormal distribution approximately, the statistical cure rates for 40 cancer sites were found to match the actuarial long-term survival rates estimated by the Kaplan-Meier method within six percentage points, at threshold year. For the two remaining cancer sites: breast and thyroid, the values of their threshold years were so long that the cancer-specific survival rates could not be obtained because the SEER data up to 1999 do not provide sufficient long-term follow-up. Table 2 shows the comparison of the cancer-specific survival rates at τ year and at long-term follow-up.

Breast cancer was further analyzed according to accepted prognostic factors. The corresponding threshold years according to stages in the SEER classification: localized (age < 50), regional (age < 50) and distant were 30.8, 27.9, and 13.1 years respectively. For histologic types: medullary, ductal combined with adenocarcinoma not otherwise specified (NOS), and lobular, the threshold years were 17.0, 30.6, and 46.0 years respectively. The threshold years according to breast tumor grades were 39.8, 26.3, and 20.7 years for Grades I, II, and III+IV respectively. The breast cancer-specific survival rates at their threshold years were 9% for distant stage, 68% for medullary histology, and 40% for Grades III+IV breast cancer. These three survival rates were only one percentage point higher when compared with the available long-term actuarial survival rates. For regional stage and Grade II breast cancer, their threshold years were 27.9 and 26.3 respectively. It can be predicted that a few more years of follow-up are needed to see the plateau and their statistical cure rates are close to 39% and 47% respectively. The most recently available SEER database is now up to end of 2001. With 2 additional years of follow-up from the end of 1999, the cancer-specific survival rates were 39% and 44% respectively. For those with τ values longer than 27 years, the cancer-specific survival rates could not be obtained because the SEER data to date do not provide sufficiently long enough follow-up time.

## Discussion

The cancer-specific survival has not included the deaths due to other causes. The cancer-specific rates also depend on the reliability of the assignment of the cause of death. Generally, cancer-specific death rates underestimate the mortality associated with a diagnosis of the specific cancer, because some patients died of other causes[26]. SEER is a set of geographically defined, population-based, central cancer registries in the United States, operated by local non-profit organizations under contract to the National Cancer Institute (NCI). Registry data are submitted electronically to the NCI on a biannual basis, and the NCI makes the data available for analysis. The SEER Program is considered the standard for quality among cancer registries around the world. Quality control has been an integral part of SEER since its inception. Every year, studies are conducted in SEER areas to evaluate the quality and completeness of the data being reported.

Gamel and Vogel [27] have compared the advantage of lognormal distribution over other distributions such as Weibull and log logit.

The 1973–1992 data were used so that the data were not out-dated. For a lognormal distribution of the survival time of those patients died of the specific cancer, there were only a very small proportion dying at the tail of follow-up (Figure 1), so it would not cause much change to the lognormal distribution, even with only 7 years of follow-up to 1999 at the tail. For those cancer sites with threshold years longer than 24 years, the 1973–1977 data were used so as to allow a long enough time for follow-up to 1999.

For 42 sites in the SEER database, the survival times of cancer patients who died of their disease followed different lognormal distributions. For 40 cancer sites, the ultimate cure rate can be roughly estimated from the cancer-specific survival rates at τ years. These are the required minimum number of follow-up years for the estimation of the cure rates. They are different for different cancer sites. For pancreatic cancer, with its typically short natural history, the cure rate can be estimated after only 2.6 years. For cancers with a longer natural history, longer follow-up periods are required; such as breast (36.2 years). These long periods are cancer-specific survival times and in reality patients may die from intercurrent non-cancer causes before τ years. For thyroid cancer, the estimated threshold year was 134.1 years. It seems that for some slow proliferating cancer types, the cure can never be estimated due to the limit of human lifetime.

We also find that the required minimum number of years of follow-up, τ, is independent of cure rate (correlation coefficient of determination, R^2 ^= 0.10). Even for cancer sites where the cure rates were >50%, the required follow-up time τ could be less than 10 years. On the other hand, for other cancer sites, the cure rates could be < 50%, and the required follow-up time were >10 years. It shows that 5- or 10-year survival rates are inadequate to reflect the statistical cure rates.

If there are more patients dying due to other causes than dying due to the specific cancer, then the cause specific survival time distribution will not be lognormal. Hence it is not expected that all cancer-specific survival time distributions will follow lognormal distributions.

According to the bell-shaped property of a normal distribution, from 0 to τ year covers 97.75% of the lognormally distributed survival time of those cancer patients who died from their specific cancer. The cancer-specific survival rates estimated at τ years, generally, slightly overestimate the long-term cure rates compared to the Kaplan-Meier method, but the differences are reasonably small, by less than six percentage points as verified empirically. We still need to follow the patients to τ year to know the actual value of the estimated cure rates.

For both rapidly and slowly proliferating cancers, we have shown that the statistical cure rates can be estimated before a stable plateau is reached in the Kaplan-Meier survival curve. It may take decades to see a stable plateau, during this waiting time many patients might be lost to follow-up or die of intercurrent diseases.

Gamel and Vogel [28] used cause-specific survival and relative survival to determine actuarial survival in breast cancer patients from the SEER database. They found that there was only minimal deviation between the two survival methods.

Ries *et al*. [29] reported up to 20-year relative survival rates (RSR) from 9 registries of the SEER database. For cancers of pancreas, esophagus and stomach, the RSR were slightly decreased after five years since diagnosis. These are consistent with the threshold years of the present study varying from 2.6 years for pancreas, to 3.9 years for esophagus, and to 5.8 years for stomach. Dickman *et al*. [30] reported similar results with 10-year RSR for the Finnish Cancer Registry. Talback *et al*.[31] also showed the similar results on two RSR graphs up to 30 years for pancreas and stomach for the Swedish Cancer Registry.

The threshold year of statistical cure for ovarian cancer was 10.4 years. It was consistent with the results of Ries *et al*. and Dickman *et al*. up to 10 years. After 10 years, there was only a slight decrease in RSR as reported by Ries *et al*. Talback *et al*. showed the same results on a RSR graph for ovarian cancer. Their results were also consistent with the present study for the cancer sites of lung, colon and skin melanomas, which have threshold years of 9.0, 12.2 and 18.2 years respectively. These consistencies show that the results obtained from two SEER registries in the present study are similar to those from 9 registries and from the Finnish and Swedish Cancer Registries.

For prostate cancer, the threshold year for statistical cure was 24.6 years. The RSR graph started to level after 24 years since diagnosis in the article of Talback *et al*.

For breast cancer, the threshold year was 36.2 years, RSR leveling was not seen even 30 years after diagnosis in the article of Talback *et al*. Leveling of RSR in breast cancer was also not seen in two separate studies by Schairer *et al*. [32] and Brenner and Hakulinen [33]. Kerr *et al*. [34] reported that the ratio of observed to expected mortality remained significantly greater than unity for at least 25 years following diagnosis and treatment, indicating a failure to demonstrate cure of the disease in a statistical sense for a median of 32 years of follow-up.

## Conclusion

The present study suggests a certain threshold year is required to wait before the statistical cure rate can be estimated for different cancer sites. Although the often used 5- or 10-year survival rates may adequately reflect statistical cure rates for cancers with short natural history, such as pancreatic cancer, this is not the case for many other cancers. This highlights the need for continued long-term cancer surveillance, especially for cancers with long natural histories such as thyroid cancer and early stage, well-differentiated breast cancer. This study is relevant for public health and cancer control. Whether knowledge of the threshold year will have any impact on decisions regarding therapy for cancers thought to have a good prognosis remains to be investigated.

## Competing interests

The author(s) declare that they have no competing interests.

## Authors' contributions

PT: Data analysis and writing of the manuscript.

EY, GCs, GV, MR, IK, VVH: Critical appraisal of the manuscript.

All authors read and approved the final manuscript.

## Pre-publication history

The pre-publication history for this paper can be accessed here:



## Supplementary Material

Additional File 1 Appendix Click here for file

## Figures and Tables

**Table 1:** List of cancer sites following lognormal distribution and their S (standard deviation of the log-survival), multiplicative standard deviation, M (mean of the log-survival), and median values at minimum chi-square with maximum P-value

Site	S	SD*	M†	Median‡	Pt. No.	P-value
Pancreas	0.45	2.82	0.60	3.98	11185	0.57
Gallbladder	0.54	3.47	0.52	3.31	1196	0.60
Liver	0.62	4.17	0.41	2.57	1701	0.22
Esophagus	0.44	2.75	0.80	6.31	4915	0.35
Hypopharynx	0.41	2.57	0.96	9.12	298	0.57
Stomach	0.51	3.24	0.82	6.61	8608	0.13
Intrahepatic Bile Duct	0.61	4.07	0.63	4.27	105	0.50
Tonsil	0.46	2.88	1.02	10.47	137	0.70
Ureter	0.41	2.57	1.15	14.13	125	0.65
Tongue	0.47	2.95	1.05	11.22	684	0.44
Brain	0.64	4.37	0.74	5.50	2870	0.16
Lung & Bronchus (age<60)	0.63	4.27	0.78	6.03	18913	0.62
Floor of Mouth	0.42	2.63	1.20	15.85	126	0.95
Small Intestine	0.55	3.55	0.99	9.77	364	0.44
Ovary	0.48	3.02	1.13	13.49	4533	0.58
Vagina	0.49	3.09	1.13	13.49	108	0.30
Cecum	0.52	3.31	1.08	12.02	2702	0.16
Nasopharynx	0.46	2.88	1.19	15.49	151	0.90
Corpus Uteri	0.45	2.82	1.22	16.60	1446	0.73
Cervix Uteri	0.46	2.88	1.21	16.22	2256	0.72
Large Intestine, NOS	0.61	4.07	0.91	8.13	622	0.72
Splenic Flexure	0.48	3.02	1.19	15.49	506	0.43
Other Nervous System#	0.50	3.16	1.14	13.80	31	0.88
Colon (age<60)	0.45	2.82	1.26	18.20	2868	0.49
Hepatic Flexure	0.55	3.55	1.06	11.48	455	0.42
Penis	0.61	4.07	0.97	9.33	107	0.39
Uterus, NOS	0.62	4.17	0.96	9.12	81	0.40
Anus, Anal Canal & Anorectum	0.44	2.75	1.33	21.38	129	0.87
Nasal Cavity, Middle Ear & Accessory Sinuses	0.52	3.31	1.20	15.85	109	0.58
Soft Tissue (with heart)	0.50	3.16	1.25	17.78	445	0.52
Bones & Joints	0.54	3.47	1.21	16.22	208	0.78
Larynx	0.50	3.16	1.30	19.95	1752	0.43
Skin Melanomas	0.42	2.63	1.49	30.90	1744	0.24
Appendix	0.48	3.02	1.40	25.12	49	0.64
Vulva	0.62	4.17	1.15	14.13	235	0.11
Retroperitoneum	0.69	4.90	1.00	10.00	100	0.59
Pleura	0.75	5.62	0.90	7.94	47	0.47
Eye & Orbit	0.43	2.69	1.59	38.90	106	0.88
Prostate (age<60, 1973–77)	0.40	2.51	1.67	46.77	413	0.44
Salivary Gland (1973–77)	0.58	3.80	1.33	21.38	65	0.81
Breast (age<60, 1973–77)	0.47	2.95	1.71	51.29	4047	0.23
Thyroid (1973–77)	0.97	9.33	1.28	19.05	123	0.15

The order of organ sites follows Table 2 in ascending order of the threshold years;

SD* Mutiplicative standard deviation of the lognormal distribution;

† M values in log-months;

‡ Median in months;

Pt. No. denotes the number of patients died of the specific cancer;

^# ^Nervous system other than brain.

**Table 2:** List of cancer sites following lognormal distribution in ascending order of τ (the threshold year for estimation of statistical cure rate)

Site	τ (yr.)	CSSR (τ)	CSSR (LT)	Diff.	Pt. No.
Pancreas	2.6	7.1	3.3	3.8	14926
Gallbladder	3.3	24.6	20.1	4.5	2123
Liver	3.7	13.7	10.6	3.1	3244
Esophagus	3.9	14.5	9.9	4.6	7021
Hypopharynx	5.1	66.9	62.2	4.7	1869
Stomach	5.8	27.3	22.3	5.0	15310
Intrahepatic Bile Duct	5.9	23.7	20.3	3.4	327
Tonsil	7.4	82.5	79.8	2.7	1137
Ureter	7.8	86.2	85.3	0.9	1122
Tongue	8.0	61.0	56.1	4.9	2264
Brain	8.6	24.4	22.0	2.4	5019
Lung & Bronchus (age<60)	9.0	17.0	11.2	5.8	24408
Floor of Mouth	9.3	80.3	75.1	5.2	1430
Small Intestine	10.3	65.7	65.2	0.5	1633
Ovary	10.4	38.0	34.9	3.1	8400
Vagina	10.6	81.0	81.0	0	618
Cecum	10.8	53.4	48.5	4.9	6766
Nasopharynx	10.8	55.4	49.5	5.9	456
Corpus Uteri	11.1	99.0	98.3	0.7	14894
Cervix Uteri	11.2	69.7	68.2	1.6	8166
Large Intestine, NOS	11.2	41.5	37.8	3.7	1466
Splenic Flexure	11.6	49.5	47.2	2.3	1223
Other Nervous System#	11.7	85.8	83.6	2.1	293
Colon (age<60)	12.2	60.1	57.1	3.0	7556
Hepatic Flexure	12.3	52.0	49.2	2.8	1184
Penis	13.0	83.7	83.7	0	766
Uterus, NOS	13.4	70.5	70.5	0	423
Anus, Anal Canal & Anorectum	13.5	85.3	85.3	0	1254
Nasal Cavity, Middle Ear & Accessory Sinuses	14.6	65.2	65.2	0	449
Soft Tissue (with heart)	15.1	69.3	66.1	3.2	1938
Bones & Joints	16.2	63.8	63.3	0.5	728
Larynx	16.8	73.7	68.6	5.1	8899
Skin Melanomas	18.2	77.8	75.8	2.0	10905
Appendix	19.7	66.8	66.8	0	199
Vulva	20.2	84.5	83.6	0.7	1931
Retroperitoneum	20.2	74.3	74.3	0	681
Pleura	21.2	72.1	72.1	0	376
Eye & Orbit	23.8	84.4	82.7	1.7	641
Prostate (age<60, 1973–77)	24.6	36.7	33.9	2.8	809
Salivary Gland (1973–77)	25.2	73.5	73.5	0	309
Breast (age<60, 1973–77)	36.2	xxx	52.5	xxx	9549
Thyroid (1973–77)	134.1	xxx	88.7	xxx	1259

CSSR (τ) denotes cancer-specific survival rate at τ year in percent, calculated by Kaplan-Meier method;

CSSR (LT) denotes long-term cancer-specific survival rate in percent, calculated by Kaplan-Meier method up to 1999;

Diff. denotes difference between CSSR (τ) and LT CSSR in percentage point;

Pt. No. denotes number of all patients, alive or dead, with or without disease;

NOS denotes not otherwise specified;

# Nervous system other than brain;

xxx denotes data not available as at 1999.
