# Supplementary material for: Minimum follow-up time required for the estimation of statistical cure of cancer patients: verification using data from 42 cancer sites in the SEER database
Source: BMC Cancer. 2005 May 17;5:48. doi: 10.1186/1471-2407-5-48 (PMC1164404; doi:10.1186/1471-2407-5-48)
Supplement: Additional File 1 — Appendix [file 1471-2407-5-48-S1.doc]

**Appendix**

Limpert *et al*. [24] has established the multiplicative property for the median (μ^*^) and multiplicative standard deviation (σ^*^) of a lognormal distribution with data x. The median (μ^*^) of the lognormal distribution is estimated as 10^M^ (the antilog of M, if logarithms with a base of 10 is used) and M is the mean of the log (x) values. The multiplicative standard deviation (σ^*^) is estimated as 10^S^ (the antilog of S, if logarithms with a base of 10 is used) and S is the standard deviation of the log (x) values. If natural logarithms are used, the median and multiplicative standard deviation are e^M^ and e^S^, respectively.

The median (μ^*^) is a scale parameter, hence if x is expressed in different units (e.g. months or years), μ^*^ changes accordingly, but σ^*^ remains the same. μ^*^ equals 10^M^/12 in years if the survival times are in months.

For a normal distribution, the interval between mean ± standard deviation covers a probability of 68.3% of the normally distributed data, and mean ± 2 (standard deviation) covers a probability of 95.5% of the data. For a lognormal distribution, the interval between median **/* multiplicative standard deviation (i.e. between μ^*^ / σ^*^ and μ^*^ × σ^*^, the sign **/* means times or divide) covers a probability of 68.3% of the lognormally distributed data. The interval between median **/* (multiplicative standard deviation)^2^, i.e. between μ^*^ / (σ^*^ )^2^ and μ^*^  × (σ^*^ )^2^, covers a probability of 95.5% of the lognormally distributed data.

If τ is equal to μ^*^ × (σ^*^ ) ^2^, then from 0 to τ year, it covers 97.75% ( =95.5% + 2.25% which is on one side of the two tails) of the lognormally distributed data which are the survival times of those cancer patients deceased from the specific cancer. When M, mean of the log (survival time), and S, standard deviation of the log (survival time), are obtained from the minimum chi-square test, μ^*^ and σ^*^ can be calculated. We define the threshold year, τ, by μ^*^ × (σ^*^ )^2^.

The term, threshold year, defined in this way means the follow-up time for patients dying from the specific cancer covers most of the survival data, leaving less than 2.25% uncovered after the threshold year. This is close enough to cure from that specific cancer. Hence the cancer-specific survival rate of the cancer patients at this time τ is almost equal to the statistical cure rate. (Figures 1 and 2)
